# Age-Dependent Patient and Trauma Characteristics and Hospital Resource Requirements—Can Improvement Be Made? An Analysis from the German Trauma Registry

**DOI:** 10.3390/medicina57040330

**Published:** 2021-04-01

**Authors:** Franziska Ziegenhain, Julian Scherer, Yannik Kalbas, Valentin Neuhaus, Rolf Lefering, Michel Teuben, Kai Sprengel, Hans-Christoph Pape, Kai Oliver Jensen, The TraumaRegister DGU

**Affiliations:** 1 Department of Trauma, University Hospital Zurich, 8091 Zurich, Switzerland; julian.scherer@usz.ch (J.S.); yannik.kalbas@usz.ch (Y.K.); valentin.neuhaus@usz.ch (V.N.); michel.teuben@usz.ch (M.T.); kai.sprengel@usz.ch (K.S.); hans-christoph.pape@usz.ch (H.-C.P.); kaioliver.jensen@usz.ch (K.O.J.); 2 Institute for Research in Operative Medicine (IFOM), University of Witten/Herdecke, 51109 Cologne, Germany; rolf.lefering@uni-wh.de; 3 Committee on Emergency Medicine, Intensive Care and Trauma Management (Sektion NIS) of the German Trauma Society (DGU), 10623 Berlin, Germany

**Keywords:** geriatric trauma, interdisciplinary, polytrauma, epidemiology

## Abstract

*Background and objectives:* The burden of geriatric trauma patients continues to rise in Western society. Injury patterns and outcomes differ from those seen in younger adults. Getting a better understanding of these differences helps medical staff to provide a better care for the elderly. The aim of this study was to determine epidemiological differences between geriatric trauma patients and their younger counterparts. To do so, we used data of polytraumatized patients from the TraumaRegister DGU^®^. *Materials and Methods:* All adult patients that were admitted between 1 January 2013 and 31 December 2017 were included from the TraumaRegister DGU^®^. Patients aged 55 and above were defined as the elderly patient group. Patients aged 18–54 were included as control group. Patient and trauma characteristics, as well as treatment and outcome were compared between groups. *Results:* A total of 114,169 severely injured trauma patients were included, of whom 55,404 were considered as elderly patients and 58,765 younger patients were selected for group 2. Older patients were more likely to be admitted to a Level II or III trauma center. Older age was associated with a higher occurrence of low energy trauma and isolated traumatic brain injury. More restricted utilization of CT-imaging at admission was observed in older patients. While the mean Injury Severity Score (ISS) throughout the age groups stayed consistent, mortality rates increased with age: the overall mortality in young trauma patients was 7.0%, and a mortality rate of 40.2% was found in patients >90 years of age. *Conclusions:* This study shows that geriatric trauma patients are more frequently injured due to low energy trauma, and more often diagnosed with isolated craniocerebral injuries than younger patients. Furthermore, utilization of diagnostic tools as well as outcome differ between both groups. Given the aging society in Western Europe, upcoming studies should focus on the right application of resources and optimizing trauma care for the geriatric trauma patient.

## 1. Introduction

Demographics in the Western world are shifting, and within the next 40 to 60 years the population of elderly people over the age of 60 years will double from 11% to 22% of the world’s population [1]. In Germany, the percentage of inhabitants aged over 67 years will rise from 16% nowadays to approximately 25% in 2040 [2]. In addition, elderly people are expected to be more active [3]. As a direct consequence, trauma specialists all over the world are confronted with a rising burden of elderly patients [4]. Studies suggest that trauma mechanisms and injury patterns in elderly people differ markedly with those seen in younger patients [5,6,7,8,9]. Literature shows that elderly trauma patients require different care compared to their younger counterparts [10,11,12,13,14]. Moreover, adverse outcome is more frequently seen in geriatric patients and associated with a higher Frailty Index [12,15,16]. Efforts have been made over the last years to reduce mortality in geriatric trauma patients [17,18,19]. The current study was designed to gain better understanding of characteristics of geriatric polytrauma and its related outcome. Hence, we aimed to identify specific patient and trauma characteristics, treatment algorithms and related outcomes in geriatric trauma patients compared to young trauma patients in this cohort.

## 2. Materials and Methods

### 2.1. The TraumaRegister DGU^®^

The TraumaRegister DGU^®^ of the German Trauma Society (Deutsche Gesellschaft für Unfallchirurgie, DGU) was founded in 1993. The aim of this multi-center database is a pseudonymized and standardized documentation of severely injured patients.

Data are collected prospectively in four consecutive time phases from the site of the accident until dis- charge from hospital: (A) Pre-hospital phase, (B) Emergency room and initial surgery, (C) Intensive care unit and (D) Discharge. The documentation includes detailed information on demographics, injury pattern, comorbidities, pre- and in-hospital management, course on intensive care unit, relevant laboratory findings including data on transfusion and outcome of each individual. The inclusion criterion is admission to hospital via emergency room with subsequent ICU/ICM care or reach the hospital with vital signs and die before admission to ICU.

The infrastructure for documentation, data management, and data analysis is provided by AUC—Academy for Trauma Surgery (AUC—Akademie der Unfallchirurgie GmbH), a company affiliated to the German Trauma Society. The scientific leadership is provided by the Committee on Emergency Medicine, Intensive Care and Trauma Management (Sektion NIS) of the German Trauma Society. The participating hospitals submit their data pseudonymized into a central database via a web-based application. Scientific data analysis is approved according to a peer review procedure laid down in the publication guideline of TraumaRegister DGU^®^.

The participating hospitals are primarily located in Germany (90%), but a rising number of hospitals of other countries contribute data as well (at the moment from Austria, Belgium, China, Finland, Luxembourg, Slovenia, Switzerland, The Netherlands, and the United Arab Emirates). Currently, approx. 33,000 cases from more than 650 hospitals are entered into the database per year.

Participation in TraumaRegister DGU^®^ is voluntary. For hospitals associated with the TraumaNetzwerk DGU^®^, however, the entry of at least a basic data set is obligatory for reasons of quality assurance [20].

The present study is in line with the publication guidelines of the TR-DGU and registered as project ID 2017-047.

We included patients aged 18 years or older, who were primarily treated by or secondarily transferred to a trauma center in Germany between 1 January 2013 to 31 December 2017 with a maximum abbreviated injury scale (mAIS) severity of not less than 3 points [21]. Data sets of the patients had to be complete and included basic information like age and sex, information on trauma mechanism (car, motorcycle, bicycle, pedestrian, falls >and <3 m, others) and injury pattern, ASA score as well as information on obtained diagnostics and specifics on the hospital stay [22]. We utilized coagulopathy at admission and the ASA classification to determine pre-existing conditions or comorbidities. The use of anticoagulant medication before the accident was collected since 2016 (Aspirin, Direct oral anticoagulants (DOAK), Heparin, Vitamin K Antagonists, Anti-platelet drugs) and used for further investigations.

Patients with an isolated femoral neck fracture were excluded. For analysis, patients were divided into two groups. The first group (EP = elderly patients) consisted of all patients with an age of 55 years or more. The second group (YP = young patients) included patients between the age of 18 and 54 years and was used as a reference group. We then further divided the first group into subgroups of 5-year clusters. Furthermore, we analyzed the injury pattern regarding the body regions with an AIS over 3. To closely evaluate the influence of a traumatic brain injury and concomitant injuries we distinguished between patients without traumatic brain injury (TBI), TBI and other injuries, and patients with isolated TBI.

### 2.2. Statistical Analysis

Categorical data are presented as percentages where the overall sample size is given. Continuous measurements were listed as mean with standard deviation (SD). In case of skewed distributions, the median is presented in addition. Formal statistical testing was avoided due to the huge sample size. Even differences of less than 1% would formally become statistically significant although being far from relevance. Statistical analysis was performed using SPSS^®^ Version 24 (IBM Inc., Armonk, NY, USA).

## 3. Results

A total of 114,169 individuals met the inclusion criteria and were included in the study. Of those, 55,404 were included in the group of elderly patients (EP group) while 58,765 patients served as the younger control group (YP group).

### 3.1. Patient Characterisitics

#### 3.1.1. Sex Distribution

A male predominance was observed in both groups with 77.4% in the YP-group and 63.7% in the EP-group. In the subgroup of the patients aged 90 and older 67.9% of the assessed patients were female (Table 1)

#### 3.1.2. Preexisting Conditions

Higher ASA scores were found in older patients. Only 4.1% of the patients in the YP-group had an ASA score of 3 or 4. In the EP-group, high ASA scores of 3 or more were observed in 35.3% of the cases and even more frequently in the subgroup of patients with an age of 85 or higher (60.0%).

**Figure 1:**
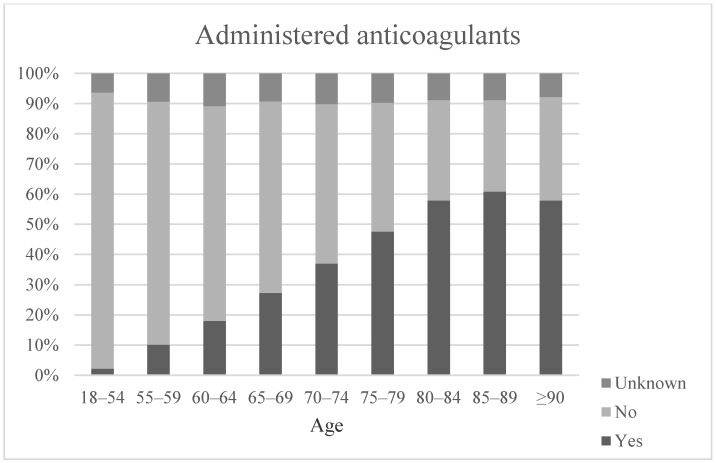
Intake of anticoagulants (Aspirin, Direct oral anticoagulants (DOAK), Heparin, Vitamin K Antagonists, Anti-platelet drugs) found by the time of admission stratified by age group.

As shown in Figure 1, the incidence of regularly administered anticoagulants was higher in the group of older trauma patients. Of 41,973 patients with a known anticoagulant-status, in mean, 23.0% were under anticoagulant medication at the time of admission (range 1.1% to 60.8%) However, coagulopathy defined as INR >1.2 or Quick <70% or PTT >40% or thrombocytes <100,000; arbitrary definition, only ranged from 15.3% to 36.3% with a mean occurrence of 21.9%.

### 3.2. Trauma Characteristics

#### 3.2.1. Trauma Mechanism

**Figure 2:**
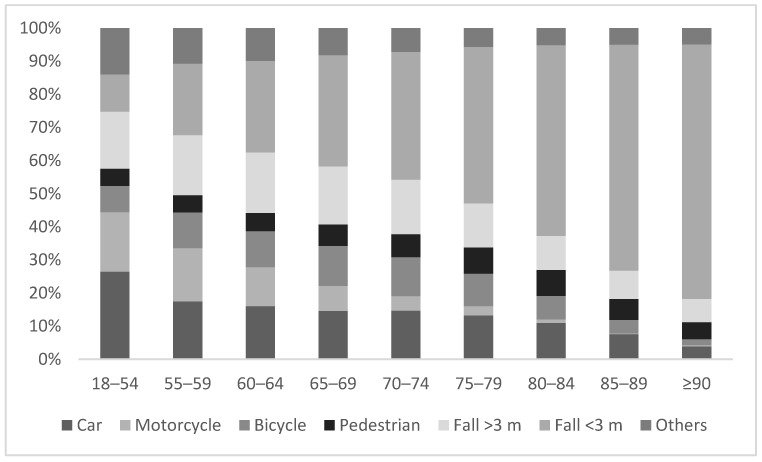
Comparison of trauma mechanism in different age groups.

Younger patients suffered mostly from road accidents with cars (26.4%) or motorcycles (18.0%). In comparison, the elderly population was found to be less likely involved in traffic accidents. In the subgroup of the over 90 years old patients, most admissions were due to low-level-falls (<3 m) (76.8%). Overall, traffic accidents made up for only 35.9% of the trauma cases in the group of elderly patients compared to 59.2% in the control group (Figure 2).

#### 3.2.2. Injury Pattern

We listed the injury pattern regarding the body regions with an AIS of three or more and the mean ISS (Injury Severity Score) of the different age groups. Results are shown in Table 2. In the control group, thoracic injuries with an AIS over 3 were most common. A reduction of serious thoracic injuries was seen compared to younger patients, with only 28.2% occurrence in the group of patients aged 90 years and older. Comparable trends were seen for abdominal injuries and injuries of the extremities. In contrast, numbers for cranial injuries with an AIS ≥ 3 increased. In the control group, 37.1% of the patients suffered from severe traumatic head injury. The highest incidence (62.6%) was shown in the group of the 85–89-year-old patients.

**Figure 3:**
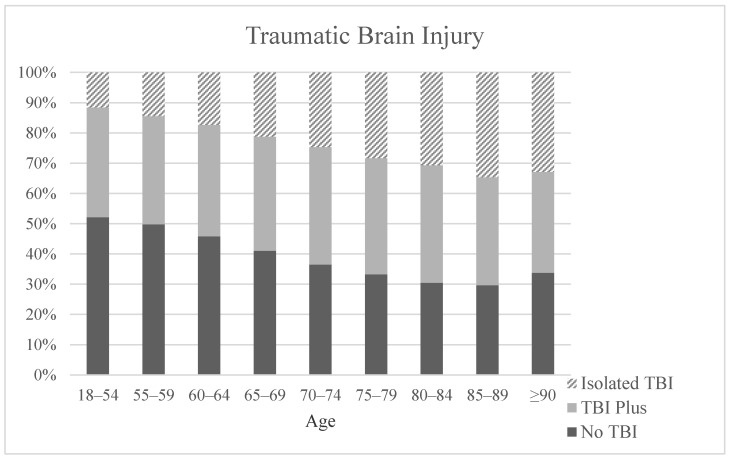
Occurrence of traumatic brain injury (TBI) depending on age.

To further evaluate the existence of traumatic brain injury and concomitant injuries we distinguished between patients without TBI (traumatic brain injury), TBI and other injuries and patients with isolated TBI. Isolated TBI applies only to very few of the patients in Group 1 (11.5%) compared to over 30% in the three subgroups of the patients aged 80 years and older. There was no difference noted in the different age groups for TBI combined with other injuries (Figure 3).

### 3.3. Allocation and Patients Triage

#### Admission

In all age groups, the majority of patients who suffered from trauma was transferred to Level I trauma centers. However, older patients were more frequently admitted to Level II or III trauma centers (37.1% of Group 1 vs. 42.8% in Group 2). Among the eldest (90 years and older), 51.7% were not treated in a Level I Trauma center.

### 3.4. Diagnostics

In primary admitted patients, whole body CT scans as well as CT scans of the head were performed less frequently in older patients. However, over 50.0% of all patients received a whole-body CT scan and more than 80% received a cranial CT scan (Table 3).

### 3.5. Outcome

#### 3.5.1. Length of Hospital Stay

The mean length of hospital-stay decreased with higher age (Table 4). The mean length of the hospital stay was shorter in deceased patients in all the assessed age groups (7.4 (deceased) vs. 19.7 (not deceased) days).

#### 3.5.2. Mortality

**Figure 4:**
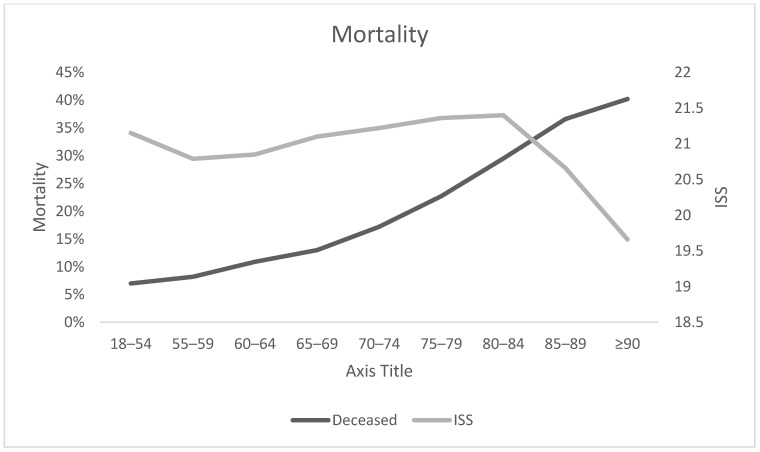
Mortality rate and ISS (Injury Severity Score) in the different age groups. Early transferred patients were excluded.

The elderly patients showed a higher mortality than the younger trauma patients. Especially the very old patients of 90 years and older had a high mortality of 40.2% (Figure 4).

## 4. Discussion

The purpose of the present study was to identify the epidemiology of elderly trauma patients. We assessed data of 114,169 patients from the TR-DGU. Several differences regarding trauma mechanism, injury pattern, performed diagnostics and outcome have been identified in this study.

The first important finding is the altered injury pattern in the group of elderly patients. The assessed data suggest that elderly people are more often admitted to the hospital due to falls from low heights and minor trauma (low-energy trauma). This was also described in several other studies [5,14,23,24]. Nevertheless, they also sustain severe, potentially live threatening, trauma.

Especially the incidence of (isolated) head injuries increases with age. Contrary to that, the control group had a lower incidence of head injuries and a higher incidence of severe extremity injuries. Priority of the trauma team should be the focused treatment of traumatic brain injuries. First of all, there is evidence of head trauma leading to an adverse outcome in geriatric trauma patients [6,25]. Furthermore, the patients in the EP group were more likely to have a coagulopathy due to pre-existing medication. In previous studies it was shown that elderly patients with traumatic brain injury and especially those with anticoagulative medication have shown to have a higher mortality [5,16]. Therefore, trauma teams should always be alerted for a possible head injury in elderly patients, especially those who receive blood thinners.

Consistent with these findings, several guidelines recommend a cranial CT scan in all geriatric patients suffering from cranial trauma [26,27]. The percentage of patients who received a cranial CT scan on admission was high in all age groups. However, we found that older patients underwent fewer cranial CT scans, which seems to be contrary to the higher incidence of traumatic brain injury and coagulopathy. Especially the very old patients were less likely to receive a cranial CT. This is surprising, as the incidence of traumatic brain injury as well as the incidence of coagulopathy was higher in the older patients group. The assessed data from the TR-DGU, therefore, may not be consistent with the general accepted guidelines [26,27]. This should be especially noted as studies have shown a better outcome after the implication of standard operating procedures [17]. Unfortunately, we were not able to assess the specific indications why a scan was not obtained in those cases. This topic should, therefore, be further investigated.

We also assessed that elderly patients underwent less whole-body CT scans. As for the head CT scans, the data does not give any explanation why the trauma team decided against a whole-body CT scan. A possible explanation is the low energy trauma mechanisms that might not call for a whole-body CT scan in the first place. The increasing incidence of isolated traumatic brain injury in elderly patients might also be a reason for the declining number of whole-body CT scans. This would explain why the number of acquired cranial CT scans stays relatively high compared to a noticeable decline in the WBCT.

Patients in the EP group were more likely to be transferred to a level II or III trauma center. This is consistent with other studies that found that elderly patients are likely to be under-triaged despite being severely injured [28]. Other authors suggest that age >70 itself should induce a trauma team activation [18]. Taking the other findings of this study into account, the preclinical triage of elderly patients, who have a high mortality after sustaining severe trauma, should be further discussed. Estimating the severity of the sustained injuries can be extremely difficult due to the altered trauma mechanisms and comorbidities of elderly trauma patients. However, established standard operating procedures for the treatment of polytraumatized patients can be advantageous for elderly patients as well [17]. Scoring systems for geriatric trauma patients to estimate the severity of the injury can be helpful in those situations [29,30].

Lastly, we found that older patients have a much higher mortality than their younger counterparts. In the group of the patients aged 90 years and older, 40.2% died after suffering from a severe trauma compared to a 7.0% mortality in the group of the younger patients. As suspected a higher ASA score was also found in the group of older patients. These findings emphasize the extreme vulnerability of geriatric trauma patients. Triage systems as well as standard operating procedures for geriatric trauma patients are required to meet the special needs of this group of patients and to reduce the incidence of adverse outcome.

## 5. Limitations

This study has several limitations. Data from registry studies in general should be carefully interpreted because there is a lack of validation of the data. Data of patients admitted to hospital without activation of the trauma team are not obtained by the TR-DGU. We also did not collect information why a CT scan was not carried out in several cases and therefore cannot make a definitive statement regarding the declining number of scans with increasing age. Data on coagulopathy was obtained, yet there are no details provided on the origins of the coagulopathy.

## 6. Conclusions

Concluding from the present analysis, elderly patients show a much higher mortality than younger patients even though the incidence of high-energy trauma appears to be lower. Adverse outcomes might be favored by co-morbidities. Severity of injury seems to be underestimated by caretakers, as geriatric patients are often not transferred to Level I trauma centers and do not receive the same amount of diagnostics. Triage systems and standard operating procedures for elderly trauma patients should be developed and put into effect to improve the outcome of this especially vulnerable patients. Focused orthogeriatric centers could provide better resource utilization.

## Figures and Tables

**Table 1:** Sex distribution within age groups.

Age Group (Years)	18–54	55–59	60–64	65–69	70–74	75–79	80–84	85–89	≥90	All
Male	77.4%	75.9%	75.4%	71.3%	65.8%	60.6%	54.9%	45.4%	32.1%	70.3%
Female	22.6%	24.1%	24.6%	28.7%	34.2%	39.4%	45.1%	54.6%	67.9%	29.7%

**Table 2:** Percentage of injuries depending on different abbreviated injury scale (AIS) body-regions separated for different age groups, ISS (Injury Severity Score) mean (SD).

	Age Groups (Years)	18–54	55–59	60–64	65–69	70–74	75–79	80–84	85–89	≥90	All
	N	55,404	9734	8419	6856	8033	9830	7721	5418	2754	114,169
**Body Regions, AIS ≥ 3**	Head	37.1%	38.8%	43.0%	48.1%	53.3%	57.2%	60.0%	62.6%	58.6%	44.5%
	Thorax	50.1%	54.2%	52.0%	48.7%	45.6%	41.0%	37.4%	29.9%	28.2%	47.1%
	Abdomen	15.5%	11.4%	10.0%	8.9%	7.0%	6.2%	4.8%	3.5%	3.1%	11.4%
	Extremities	33.6%	27.5%	25.8%	23.6%	21.8%	21.4%	22.0%	24.6%	28.4%	28.7%
	ISS	21.15 (11.6)	20.79 (11.1)	20.85 (10.7)	21.10 (10.9)	21.22 (10.9)	21.36 (10.9)	21.40 (11.1)	20.66 (10.9)	19.66 (10.6)	21.07 (11.2)

**Table 3:** Percentage of primary admitted patients who received a whole-body CT scan (WBCT)/cranial CT scan (CCT).

Age Group (Years)	18–54	55–59	60–64	65–69	70–74	75–79	80–84	85–89	≥90
WBCT	84.5%	82.1%	81.7%	78.7%	76.1%	72.6%	67.8%	60.6%	52.5%
CCT	91.8%	91.6%	91.8%	91.9%	91.5%	91.5%	89.9%	87.1%	81.7%

**Table 4:** Length of hospital stay separated in different age groups and survivors/non-survivors. Early transferred patients (48 h) were excluded.

Age Group	Survivor		Non-Survivor	
	*n*	Median/Mean (SD)	*n*	Median/Mean (SD)
18–54	47,547	14/19.4 (19.2)	3567	2/5.9 (11.8)
55–59	8270	15/20.3 (20.1)	734	3/7.9 (12.3)
60–64	6897	15/20.6 (19)	844	3/8.2 (14.1)
65–69	5527	16/20.7 (18.8)	825	4/8.6 (14.8)
70–74	6132	16/21.1 (18.6)	1276	4/8.2 (12.5)
75–79	6978	16/20.5 (17.2)	2050	3/8.5 (12.7)
80–84	5051	16/19.6 (15.1)	2112	3/8 (11.8)
85–89	3213	14/17.6 (14.1)	1854	3/7.4 (10.2)
≥90	1563	12/14.7 (10.4)	1051	3/6.4 (12.2)
ALL	91178	15/19.7 (18.6)	14313	3/7.4 (12.2)

## Data Availability

The analyzed datasets during the current study are available from the corresponding author on reasonable request.
